# The effect of patient characteristics on variability in pain and function over two years in early knee osteoarthritis

**DOI:** 10.1186/1477-7525-3-59

**Published:** 2005-09-27

**Authors:** Przemyslaw T Paradowski, Martin Englund, L Stefan Lohmander, Ewa M Roos

**Affiliations:** 1 Department of Orthopedics, Lund University, Lund University Hospital, SE-221 85 Lund, Sweden; 2 Department of Reconstructive Surgery and Artrhroscopy of the Knee Joint, Medical University, Drewnowska 75, 91-002 Lodz, Poland

## Abstract

**Background:**

Large variations in pain and function are seen over time in subjects at risk for and with radiographic knee osteoarthritis (OA). We hypothesized that this variation may be related not only to knee OA but also to patient characteristics. The objective of this study was to investigate the influence of age, gender, and body mass index (BMI) on clinically relevant change in pain and function over two years in subjects at high risk for or with knee OA.

**Methods:**

We assessed 143 individuals (16% women, mean age 50 years [range 27–83]) twice; 14 and 16 years after isolated meniscectomy. Subjects completed one disease-specific questionnaire, the Knee injury and Osteoarthritis Outcome Score (KOOS) and one generic measure, the SF-36. Individuals with a BMI between 25 and 29.9 were considered overweight, while individuals with a BMI of 30 or more were considered obese.

**Results:**

Subjects aged 46–56 (the middle tertile) were more likely to change (≥10 points on a 0–100 scale) in the KOOS subscale Activities of Daily Living (ADL) than younger subjects (odds ratio [OR] 4.5, 95% confidence interval [95% CI] 1.5–13.0). Essentially the same result was obtained after adjusting for baseline values. Overweight or obesity was a risk factor for clinically relevant change for knee pain (OR 2.4, 95% CI 1.0 – 5.8, OR 4.0, 95% CI 1.2 – 13.6) and obesity for change in ADL (OR 4.3, 95% CI 1.2 – 15.4). The results did not remain significant when adjusted for the respective baseline value. Being symptomatic was strongly associated with increased variation in pain and function while presence or absence of radiographic changes did not influence change over two years in this cohort.

**Conclusion:**

In a population highly enriched in early-stage and established knee OA, symptomatic, middle-aged, and overweight or obese subjects were more likely to vary in their knee function and pain over two years. The natural course of knee pain and function may be associated with subject characteristics such as age and BMI.

## Background

The osteoarthritis (OA) disease process begins much earlier than radiographic changes can be detected on plain radiographs. Individuals with such incipient OA may represent an attractive target group for future therapy aimed at slowing or stopping the further progression of OA. Individuals that have undergone meniscectomy constitute a high risk group for development of knee OA, and may represent a suitable group for studies on OA progression, as well as clinical trials in OA [1-3]. In a previous study, we investigated the natural variation in symptoms in a cohort of patients who had undergone meniscectomy 14–16 years earlier, and found that one in three patients reported a clinically relevant change in pain and function over two years [4]. Variability of symptoms in either direction is of interest to understand the natural course of knee OA. We hypothesized that the natural variation of symptoms over time may not only depend on factors related to the knee (such as the presence of joint pathology), but also on factors not directly related to the knee, e.g. patient characteristics such as age and weight. The objective of this study was to investigate the influence of age, gender, and body mass index (BMI) on clinically significant variation in pain and function over two years in subjects who had undergone meniscectomy several years before. We used a knee-specific instrument as the primary outcome measure and a generic questionnaire as a secondary tool.

## Methods

### Patients

Approval was obtained from the Research Ethics Committee at the Faculty of Medicine, Lund University, Sweden. All patients recruited were identified by searching the surgical records at the Department of Orthopedics, Lund University Hospital, Sweden [4]. A total of 552 subjects had undergone meniscectomy between 1983 and 1985. Inclusion and exclusion criteria allowed us to identify 264 patients who, in 1998, were sent self-administered questionnaires that assessed their knee-specific symptoms, knee function, and general health status. Of those, 211 replied (response rate 80%). In 2000, follow-up questionnaires were distributed to 200 subjects (eleven subjects were excluded because of death or newly-discovered co-morbidity). Replies were received from 146 individuals (73%). Three patients completed only one of two different questionnaires. The remaining 143 patients (84% men) formed the study group. Neither the baseline scores nor patient characteristics for the non-responders differed from the subjects completing the questionnaires at both time points. Self-reported weight and height was obtained from 141 of the patients at the second evaluation. Individuals with a BMI between 25 and 29.9 were considered overweight, while individuals with a BMI of 30 or more were considered obese. The subjects' mean age at the first follow-up was 50 (range 27–83) years. Assessments were carried out with a median interval of 2.3 (range 2.3 to 3.0) years.

### Disease-specific questionnaire

The Knee injury and Osteoarthritis Outcome Score (KOOS) Swedish version LK 1.0 was used [5]. KOOS is a 42-item self-administered knee-specific questionnaire based on the WOMAC Osteoarthritis Index [6]. KOOS was developed to be used for short- and long-term follow-up studies of knee injuries, and it comprises five subscales: Pain, other Symptoms, Activities of Daily Living (ADL), Sports and Recreation Function, and knee-related Quality of Life. A separate score ranging from 0–100, where 100 represents the best result, is calculated for each subscale. The questionnaire and scoring manual are available from the website . The KOOS is valid and reliable in follow-up of meniscectomy [5], anterior cruciate ligament reconstruction [7] and total knee replacement [8]. The patients completed the KOOS questionnaire answering questions on their operated knee.

### Generic questionnaire

The 36-item Short Form (SF-36) of the Medical Outcome Study, Swedish version was used [9]. The SF-36 is a self-administered, generic measurement tool which has been previously validated for use in the general populations [10] as well as in selected populations with knee OA [11]. Responses to 35 of 36 questions are aggregated into eight dimensions: Physical Function, Role-Physical, Role-Emotional, Bodily Pain, Social Functioning, Mental Health, Vitality, and perception of General Health. Responses vary from dichotomous (yes/no) to a six-point verbal rating scale. Results are calculated and presented as a profile of scores of each of eight dimensions.

### Clinically important variation

The minimal perceptible clinical improvement (MPCI) represents the difference on the measurement scale associated with the smallest change in the health status that could be detected by the patient. Since the KOOS questionnaire contains the full and original version of the WOMAC index and WOMAC scores can easily be calculated, we used the MPCI as described for the WOMAC subscales pain, physical function, and stiffness [12]. According to this standard a level of 10 points or more on a 0–100 scale was established as a cut-off representing a clinically significant difference. We applied the same cut-off for the generic SF-36 outcome measure as for the KOOS.

### Definition of a symptomatic index knee

Since there is no agreement upon a cut-off with regard to 'symptomatic' in this context, we used a previously applied definition based on the patient's self-report from the KOOS questionnaire [2]. This operational definition aimed at identifying individuals symptomatic enough to possibly seek medical care. The definition required that the value of the KOOS subscale QOL *and *two out of the four additional subscales should be equal to or less than the score obtained as follows: At least 50% of the questions within the subscale were answered with at least one step decrease from the best response (indicating no pain/best possible function etc.) on a 5-point Likert scale. After conversion to a 0–100 worst to best scale the cut-offs were as follows: Pain ≤ 86.1, Symptoms ≤ 85.7, ADL ≤ 86.8, Sport/Rec ≤ 85.0 and QOL ≤ 87.5.

### Radiographic examination

One-hundred and thirty-three (93%) of the subjects had undergone radiographic knee examination at the second assessment. The radiographic technique and the grading of radiographs have been detailed [3]. The cut-off for radiographic OA corresponded to grade 2 or worse on the Kellgren and Lawrence scale [13].

### Statistics

Logistic regression models, both unadjusted and adjusted for baseline values, were used to evaluate the association of each study variable with clinically relevant change. The logistic regression model included age divided into tertiles, gender, and body mass index (categorized). The odds ratio (OR) estimates with 95% confidence intervals (95% CIs), and results from the likelihood ratio test, expressed as *P*-values, were based on the models. We considered a *P*-value of 0.05 or less significant, and all tests were two-sided (SPSS for Windows release 12.0.1, SPSS Inc.). No prior sample size determination was made due to the observational character of the present study. However, in a binomial post hoc power calculation, using prevalence estimates and numbers from the existing dataset (n = 143, clinical relevant change occurring in 8 of 50 subjects in the reference category, and at a significance level of 0.05), we would have 80% power to detect an OR of 3.16

## Results

### Age

**Figure 1:**
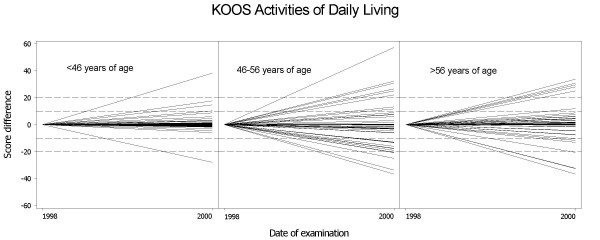
**Variability in the subscale Activities of Daily Living of the Knee injury and Osteoarthritis Outcome Score (KOOS) over two years**. Subjects are divided into three groups (tertiles) according to their age. Each line represents one individual visualizing the difference between the score in 1998 (presented as zero, left endpoint of line) and in 2000 (right endpoint of the same line).

There was an association of age 46–56 (middle tertile) and clinically relevant change (≥10 points on a 0–100 scale) for the KOOS subscale ADL (OR 4.5, 95% CI 1.5 – 13.0) when unadjusted for baseline (figure 1, table 2), but otherwise there were no significant associations detected with respect to age. The association between the intermediate age tertile and ADL remained significant after adjusting for baseline values, i.e., the subjects' ADL values from 1998 (table 2).

**Table 2:** The effect of demographic characteristics on clinically relevant variation in *function *over two years

	**KOOS Activities of Daily Living**					**SF-36 Physical Function**				
Characteristic	Prevalence of clinically relevant change		Unadjusted for baseline values	Adjusted for baseline values		Prevalence of clinically relevant change		Unadjusted for baseline values	Adjusted for baseline value	
	N	%	OR	OR	95% CI	N	%	OR	OR	95% CI

Age										
<46†	6/49	12				21/49	43			
46–56	19/47	40	4.5	3.8	1.2–12.4	24/45	53	1.2	0.9	0.3–2.4
>56	13/47	28	2.5	1.8	0.5–6.2	23/46	50	1.3	0.5	0.2–1.4
Gender										
men†	32/120	27				54/118	46			
women	6/23	26	1.2	0.7	0.2–2.3	14/22	64	2.4	1.9	0.6–6.1
BMI (kg/m^2^)										
<25†	9/61	15				21/60	35			
25–29.9	22/64	34	1.4	2.0	0.5–3.9	39/63	62	3.2	2.4	1.0–5.7
≥30	7/16	44	1.4	1.8	0.3–6.3	6/15	40	1.2	0.1	0.2–0.9

KOOS = Knee injury and Osteoarthritis Outcome Score, OR = odds ratio, 95% CI = 95% confidence interval, BMI = body mass index.

* All risk factors listed are entered simultaneously in each model.

† Reference category.

### Gender

No significant associations between gender and clinically relevant change for pain or function were detected (tables 1, 2).

**Table 1:** The effect of demographic characteristics on clinically relevant variation in *pain *over two years*

	**KOOS Pain**					**SF-36 Bodily Pain**				
Characteristic	Prevalence of clinically relevant change		Unadjusted for baseline values	Adjusted for baseline values		Prevalence of clinically relevant change		Unadjusted for baseline values	Adjusted for baseline value	
	N	%	OR	OR	95% CI	N	%	OR	OR	95% CI

Age										
<46†	11/49	22				27/49	55			
46–56	17/47	36	1.5	1.1	0.4–3.1	32/47	68	1.2	1.1	0.4–2.8
>56	12/47	26	1.0	0.9	0.3–2.4	34/47	72	1.9	1.7	0.7–4.5
Gender										
men†	35/120	29				78/120	65			
women	5/23	22	1.2	0.5	0.2–1.8	15/23	65	1.2	1.1	0.4–3.2
BMI (kg/m^2^)										
<25†	10/61	16				34/61	56			
25–29.9	22/64	34	2.4	2.0	0.8–4.9	47/64	73	2.3	1.9	0.8–4.3
≥30	7/16	44	4.0	1.8	0.4–7.1	11/16	69	1.4	0.6	0.2–2.5

KOOS = Knee injury and Osteoarthritis Outcome Score, OR = odds ratio, 95% CI = 95% confidence interval, BMI = body mass index.

* All risk factors listed are entered simultaneously in each model.

† Reference category.

### Body mass index

In a model unadjusted for baseline, overweight or obesity was a risk factor for clinical variation in the knee-specific subscale Pain (OR 2.4, 95% CI 1.0 – 5.8, OR 4.0, 95% CI 1.2 – 13.6), and obesity for change in ADL of the KOOS questionnaire (OR 4.3, 95% CI 1.2 – 15.4). However, when the baseline values were included in the model, the associations did not remain significant (tables 1, 2). For pain in general and Physical Function (SF-36), overweight was associated with clinically relevant change (OR 2.2, 95% CI 1.0 – 4.8, OR 3.2, 95% CI 1.5 – 6.9) unadjusted for baseline. The association remained for SF-36 Physical Function after adjusting for baseline values, but not for SF-36 Bodily Pain (tables 1, 2).

### Radiographic knee OA

Fifty-eight of the 133 x-rayed subjects (44%) had radiographic tibiofemoral or patellofemoral OA in their index knee. In separate analyses (adjusted for age, gender, BMI, and unadjusted and adjusted for baseline values, respectively), we also evaluated the effect of radiographic knee OA on clinically relevant change. We found no association between the presence of radiographic OA (K/L grade ≥ 2) and clinical variation, neither in the knee-specific KOOS nor the generic SF-36 subscales (p ≥ 0.09).

### Symptomatic knee

In all the present analyses, the baseline values were highly significant, where lower (worse) score was associated with clinically relevant change (p ≤ 0.001).

According to our definition of a 'symptomatic' knee based on the KOOS questionnaire, there were 61 individuals who were symptomatic at entry and 66 who fulfilled the same criteria at the second assessment. In a model adjusted for age, gender, and BMI, the likelihood of clinically relevant variation over time for subjects who were *symptomatic *at baseline was significantly higher for all the outcomes than subjects defined as *asymptomatic*: KOOS Pain (OR 5.8, 95% CI 2.4 – 14.1), KOOS ADL (OR 4.5, 95% CI 1.9. – 11.0), SF-36 Bodily Pain (OR 3.0, 95% CI 1.3 – 6.8), and SF-36 Physical Function (OR 3.8, 95% CI 1.7 – 8.5).

## Discussion

The present study indicates that being knee-symptomatic, middle-aged, and overweight may predispose for variation in pain and function over two years. These factors are therefore relevant to take into account when deciding patient inclusion and exclusion criteria, outcome measures, and the number needed in clinical trials in subjects with early-stage knee OA.

The Bristol 'OA 500' study is one of few studies on the natural variation of symptoms in knee OA [14]. In this cohort, several baseline variables including age, gender, and BMI were analyzed as possible predictors of change in a combined index including change in pain, change in index joints, and global change. Neither age, gender, nor BMI were shown to influence the variation over eight years [14]. In comparison with our study subjects, the Bristol 'OA 500' cohort was on average 10 years older, held more women (69 vs. 16%), and included subjects with OA not only of the knee but also of the hip and hand. The mean BMI or presence of previous knee injuries was not reported.

OA is usually studied in the elderly. However, it is well recognized that OA may develop during middle-age or earlier [15,16]. The age range of 27 to 83 for our cohort allowed us to study the variation in symptoms in different age categories. We found an increased variability in knee-related function in patients aged 46–56, compared with the younger age group. Younger subjects scored well at entry, and in general their outcome remained good over time. These age-related findings may reflect the developmental phases of OA and correspond to an increase in the report of knee disability seen in the population during middle-age [16,17]. With increased age there may also be large changes in lifestyle due to early retirement or other alterations of the psychosocial situation that may affect self-reported symptoms and knee function. The oldest subgroup (aged >56) seem to form a more stable group than the aged 46–56, but low subject numbers limit interpretation of the results.

In analytic models unadjusted for baseline, overweight or obesity was a predictor of clinically relevant variation in outcome over two years. However, when adjusting for baseline scores, these associations did not remain significant. This is likely an effect of the strong association between high BMI and being symptomatic. In a closely related study we found no evidence that subjects had become sedentary due to symptoms and then obese (as cause and effect) [3].

Having a symptomatic knee, in comparison with having a non-symptomatic knee, was strongly associated with large variability over two years. We cannot exclude the contribution of a ceiling effect, i.e., an individual with KOOS Pain ≥ 91 cannot improve by 10 points. There was no floor effect possible, as no study subjects had a score of 10 or worse. The finding of symptoms being a strong predictor of change has less importance for future design in OA trials since a common inclusion criterion is at least moderate pain and functional limitations to be able to detect a clinically relevant improvement from the intervention applied.

We found no significant influence of radiographic status on variation in pain and function in this cohort, consistent with the well-known discordance between radiographic status and pain in population-based studies of OA [18,19].

We arbitrarily applied the same cut-off for clinically relevant change for the generic SF-36 as for the knee-specific measure KOOS. This serves as an important limitation in interpreting the SF-36 results. The pain and function subscales of the KOOS and SF-36 measures hold different number of items, and the different items may have different numbers of response options. A change in response of one step, e.g. from mild to moderate, will have greater impact on the final score if the scale has fewer items, or fewer response options. For the KOOS subscale Pain, a one step change in response option will result in a score change of 2.8 points. For the SF-36 subscale Bodily Pain the corresponding change in response would yield a score change of 6–20 points (depending on the item/response option changed), explaining the larger proportion of patients changing on the SF-36, compared with the KOOS.

Other limitations of our study include low subject numbers and not taking into consideration several factors in that may be important to the outcome studied, most importantly medication, co-morbidities, educational level, coping, and mood status. Also, the use of self-estimates of weight and length from the second assessment and the low proportion of women serve as limitations. Meniscectomy is more frequently performed in men [20,21], the reasons for which are not clear. We were unable to evaluate the possible influence of "regression to the mean" phenomenon as we only had data from two time points. Strengths of our study include the use of validated self-reported questionnaires which help avoid investigator bias, and the high follow-up rate of 73%.

## Conclusion

In conclusion, we report that among a group highly enriched in early-stage and established knee OA, symptoms at baseline, middle age, and overweight or obesity are factors related to clinically relevant change in pain or knee function over two years. Variation over time in knee symptoms related to OA may thus also be dependent on factors not directly related to the knee.

## Authors' contributions

EMR and LSL planned the study and collected the data. ME identified the patient cohort, collected the data, and performed the statistical analysis. PTP participated in the statistical analysis and drafted the manuscript together with ME (ME and PTP contributed equally to this study). EMR and LSL revised the manuscript. All authors read and approved the final manuscript.
